# Correlation of mitochondrial respiration in platelets, peripheral blood mononuclear cells and muscle fibers

**DOI:** 10.1016/j.heliyon.2024.e26745

**Published:** 2024-02-23

**Authors:** Emil Westerlund, Sigurður E. Marelsson, Michael Karlsson, Fredrik Sjövall, Imen Chamkha, Eleonor Åsander Frostner, Johan Lundgren, Vineta Fellman, Erik A. Eklund, Katarina Steding-Ehrenborg, Niklas Darin, Gesine Paul, Magnus J. Hansson, Johannes K. Ehinger, Eskil Elmér

**Affiliations:** a Mitochondrial Medicine, Department of Clinical Sciences Lund, Lund University, Lund, Sweden; b Emergency Department, Kungälv Hospital, Kungälv, Sweden; c Children's Medical Center, Landspitali-The National University Hospital of Iceland, Reykjavík, Iceland; d Department of Neurosurgery, Rigshospitalet, Copenhagen, Denmark; e Department of Intensive- and Perioperative Care, Skåne University Hospital, Malmö, Sweden; f Department of Pediatrics, Skåne University Hospital, Lund University, Lund, Sweden; g Clinical Physiology, Department of Clinical Sciences Lund, Skåne University Hospital, Lund University, Lund, Sweden; h Department of Pediatrics, The Queen Silvia Children's Hospital, University of Gothenburg, Gothenburg, Sweden; i Translational Neurology Group and Wallenberg Center for Molecular Medicine, Department of Clinical Sciences Lund, Lund University, Lund, Sweden; j Department of Clinical Sciences Lund, Otorhinolaryngology, Head and Neck Surgery, Skåne University Hospital, Lund University, Lund, Sweden

**Keywords:** Mitochondria, Biomarkers, Platelets, PMBCs, Muscle, Correlation

## Abstract

There is a growing interest for the possibility of using peripheral blood cells (including platelets) as markers for mitochondrial function in less accessible tissues. Only a few studies have examined the correlation between respiration in blood and muscle tissue, with small sample sizes and conflicting results.

This study investigated the correlation of mitochondrial respiration within and across tissues. Additional analyses were performed to elucidate which blood cell type would be most useful for assessing systemic mitochondrial function.

There was a significant but weak within tissue correlation between platelets and peripheral blood mononuclear cells (PBMCs). Neither PBMCs nor platelet respiration correlated significantly with muscle respiration.

Muscle fibers from a group of athletes had higher mass-specific respiration, due to higher mitochondrial content than non-athlete controls, but this finding was not replicated in either of the blood cell types. In a group of patients with primary mitochondrial diseases, there were significant differences in blood cell respiration compared to healthy controls, particularly in platelets. Platelet respiration generally correlated better with the citrate synthase activity of each sample, in comparison to PBMCs.

In conclusion, this study does not support the theory that blood cells can be used as accurate biomarkers to detect minor alterations in muscle respiration. However, in some instances, pronounced mitochondrial abnormalities might be reflected across tissues and detectable in blood cells, with more promising findings for platelets than PBMCs.

## 1 Introduction

There is growing interest in using peripheral blood cells as markers for mitochondrial function in less accessible tissues or for systemic mitochondrial function. This applies both to physiological research and clinical medicine [1,2].

Mitochondrial respiration plays an important role in physiological processes such as aging, exercise and metabolic regulation [3,4]. Naturally, there has been a tradition of studying these phenomena in organs with high energy demand and large amounts of mitochondria, especially skeletal muscle. Also in clinical medicine, where the assessment of mitochondrial respiration is currently used to diagnose primary (inherited) mitochondrial disease, skeletal muscle is the preferred tissue to sample [5].

While generally safe for a healthy adult, a muscle biopsy is an invasive procedure that has to be performed by a trained physician, requires local anesthesia and may cause pain and local soreness for a few days after [6]. This poses problems if a study requires large sample sizes or repeated measurements [7]. In clinical medicine there are additional problems associated with muscle biopsies. Mitochondrial disease is generally suspected and diagnosed in early childhood but a child – as opposed to an adult – will often need general anesthesia, or at least sedation, for the procedure [5].

A blood sample, in contrast to a muscle biopsy, can easily be obtained through a minimally invasive technique and used for mitochondrial analyses [8]. While potential practical benefits are obvious, it is still unclear how reliable blood cell respiration is as a biomarker for muscle or systemic respiration as most studies have examined this relationship indirectly.

Only a few studies have examined the direct correlation of mitochondrial respiration between muscle fibers and blood cells (including platelets, which, for the purposes of this article will be labeled ‘cells’ as opposed to ‘cell fragments’). In these studies, sample sizes have been small and the results conflicting. One study reported a strong correlation between mitochondrial respiration in monocytes and skeletal muscle in monkeys (n = 18) but weaker correlation between platelets and muscle cells [9]. A study on humans featuring young healthy men (n = 10) found no correlation between respiration in peripheral blood mononuclear cells (PBMCs), including a small fraction of monocytes, and permeabilized muscle cells [10]. Another study, where participants were a mix of healthy and diseased women (n = 32), found no correlation between respiration of permeabilized muscle fibers and intact platelets, nor intact PBMCs. The only significant correlations were found in two out of 12 tested parameters (‘CI LEAK’ and ‘OXPHOS coupling efficiency’) in a subset of the participants (n = 12–13), and only for permeabilized platelets [11]. These findings are in some sense contrasted by another study, similar in size (n = 32), that found more consistent correlations (several respiratory states correlated in the main study population) between platelets and muscle cells (but did not examine other blood cell types). However, this study differed from the former as it used intact platelets and included older participants of both sexes [12].

To our knowledge, no study has so far examined the correlation of mitochondrial respiration between different types of blood cells. Indeed, since all types of blood cells are equally accessible it would rarely be relevant in practice to use one as a biomarker for the other. But the question of correlation within blood tissue (i.e. between different blood cells or blood cell types) is still interesting for theoretical purposes. PBMCs (lymphocytes and monocytes) and platelets are being studied individually as candidate biomarkers, and it is unlikely that they both correlate highly with muscle tissue respiration unless they correlate with each other.

In this study the first aim was to examine if respiration in platelets and PBMCs correlate with one another. Some degree of within tissue correlation was assumed to be a prerequisite for finding a correlation across tissues. The within-tissue correlation was hypothesized to be higher than that across tissues, or more specifically, in the present study, between cells from blood and muscle tissue.

Accordingly, the second aim was to make an across tissue comparison to directly compare platelets and PBMCs to muscle fibers in a subgroup of healthy volunteers.

Additional analyses were made to further explore the results of the main analyses. We analyzed the correlation between respiration and mitochondrial content in the different sample types and made groupwise analyses in two subgroups, each subgroup representing a possible application area of blood cells as biomarkers and also representing two conceptually different forms of metabolic alterations. The first of these subgroups included semi-professional athletes, who were expected to have physiological, acquired alterations of their muscle respiration as a result of exercise, in comparison with non-athlete controls. We hypothesized that these alterations would appear to some degree also in blood cells. The second subgroup were pediatric patients with confirmed primary mitochondrial disease. They were hypothesized to have some degree of altered blood cell respiration due to genetic defects of the enzymes of the respiratory system, when compared to other pediatric patients without mitochondrial disease.

## 2 Results

### 2.1 Characteristics of the study population

An overview of the study population and the different subpopulations used for different analyses is presented in Table 1. In total, 318 individuals were included. Data from either PBMCs or plalelets, or both, for these individuals (but not the data from the 24 muscle biopsies) have been published before in other contexts [[13], [14], [15], [16], [17], [18], [19], [20]]. The ages ranged from 3 days to 86 years with a roughly equal sex distribution (148 females). Of the total study population, 226 participants were patients and 92 were healthy volunteers. Adult patients primarily had neurological diseases (n = 153) such as Parkinson's disease (n = 57), essential tremor (n = 18), amyotrophic lateral sclerosis (n = 16) and Huntington's disease (n = 13), and another subset were patients admitted to the ICU with sepsis (n = 14). Healthy adult participants were composed of neurologically healthy age-matched controls to the neurological cases, healthy student volunteers and semi-professional athletes [14,15,21]. There were 82 children (under the age of 18 years) in this study. Out of these, 23 were otherwise healthy patients undergoing minor elective surgery [13]. There were 59 pediatric patients, nine of whom had confirmed primary mitochondrial disease. The remaining 50 patients were acutely ill patients where mitochondrial disease was included in the differential diagnosis. Many of these turned out to have other – mainly neurological or metabolic – diseases, while others eventually recovered. In addition to blood samples, which were obtained from all participants, muscle biopsies were collected from 24 of the healthy adult participants. The muscle respiration data have not been published previously.

**Table 1:** Study population and subgroups. CS = citrate synthase SD = standard deviation *Adult defined as ≥ 18 years, child as < 18 years **Exact age was unavailable for one adult participant.

	Related table/figure	n	Female/male (not recorded)	Adults/children*	Median age in years (range); mean age in years (SD)*	Patients/healthy participants
Total study population	Table 3, Fig. 2	318	148/167 (3)	236/82	58 (0–86); 45.1 (28.4)	226/92
All healthy participants	–	92	51/38 (3)	69/23	26 (0–80); 35.0 (26.0)	0/92
All patients	–	226	116/110	167/59	63 (0–86); 49.2 (28.4)	226/0
All cases where CS activity was measured	Table 3, Fig. 2, Fig. 3, Fig. 4	162	85/74 (3)	94/68	23 (0–84); 30.1 (27.2)	78/84
All healthy particiapts where CS activity was measured	Fig. 3B	84	47/34 (3)	62/22	24 (0–80); 32.4 (24.7)	0/84
All patients where CS activity was measured	Fig. 3B	78	38/40 (−)	32/46	12.5 (0–84); 29.0 (29.8)	78/0
Cases where muscle respiration was measured, i.e. athletes (n = 5) and non-athlete controls (n = 19)	Table 3, Fig. 3A–. 4	24	10/14 (−)	24/0	24 (19–42); 25.5 (5.4)	0/24
Pediatric patients with (n = 9) and without (n = 50) mitochondrial diseae	Fig. 5	59	33/26 (−)	0/59	3 (0–17); 4.8 (5.1)	59/0

For mean blood cell respiratory data for the healthy population (n = 92) and the healthy adult population (n = 69), we refer to Supplementary Table 1.

### 2.2 Correlation within and across tissues

Table 3 and Fig. 2A summarize correlation within and across tissues. There was a general but very weak correlation between PBMCs and platelets and the correlation was stronger when normalizing for CS (mitochondrial content) or using internal ratios (calculating ratios between different respiratory rates is another way of normalizing the measurements, as the value of such ratios are independent of cellular mitochondrial content). The N/NS-pathway control ratio displayed the strongest correlation of 0.42 (95% CI 0.33–0.51, P < 0.001). The correlations between PBMCs and platelets were also tested for several subgroups of the main study population. These post hoc-analyses are presented in Supplementary Table 3 and Supplementary Table 4.

**Table 2:** Overview of respiratory rates and internal ratios. P = OXPHOS; L = LEAK; E = ET; N = NADH; S = succinate; CI = complex I; CII = complex II; CIV = complex IV; DMP = digitonin, malate, pyruvate; PM = pyruvate, malate; PGM = pyruvate, glutamate, malate; PGMS = pyruvate, glutamate, malate, succinate; TMPD = tetramethylphenylenediamine; FCCP = protonophore carbonyl cyanide 4-(trifluoromethoxy) phenylhydrazone; ADP = adenosine diphosphate *Measurements made after respective additions to the chamber during the experiment (and corrected for antimycin A) as illustrated in Fig. 1.

Respiratory rates and ratios	Abbreviation	Corresponding measurement or calculation*	Explanation
ROUTINE respiration	*R*	Routine	Routine respiration in intact cells.
Background respiration	*L*(n)	DMP	Background respiration in permeabilized cells with malate and pyruvate. This rate may alternatively be called LEAK respiration as it reflects the same coupling state as *L*(Omy) (see below) but it is labeled *L*(n) to indicate that the absence of ADP is limiting phosphorylation, as opposed to inhibition of ATP synthase for *L*(Omy).
N-OXPHOS capacity (PM)	PM_*P*_	ADP	Maximal ADP stimulated respiration with pyruvate and malate (N-pathway/CI-linked pathway).
N-OXPHOS capacity	PGM_*P*_	glutamate	Maximal ADP stimulated respiration with pyruvate, malate and glutamate (N-pathway/CI-linked pathway).
NS-OXPHOS capacity	PGMS_*P*_	succinate	Maximal ADP stimulated respiration with pyruvate, malate, glutamate and succinate (NS pathway/CI–CII-linked pathway).
LEAK respiration	*L*(Omy)	oligomycin	Background respiration (with oligomycin-inhibited ATP synthase) with pyruvate, malate, glutamate and succinate. Consists mainly of proton leak over the inner mitochondrial membrane.
NS-ET capacity	PGMS_*E*_	FCCP	Maximal noncoupled respiration with pyruvate, malate, glutamate and succinate (NS pathway/CI–CII-linked pathway).
S-ET capacity	S_*E*_	rotenone	Maximal noncoupled respiration with succinate, CI inhibited by rotenone (S-linked pathway (CII-linked).
CIV capacity	CIV_*E*_	TMPD-azide	Maximal complex IV activity
Respiratory control ratio	RCR	succinate/oligomycin	Coupling efficiency (OPXHOS capacity relative LEAK respiration)
L/E coupling control ratio	L/E CCR	oligomycin/FCCP	Intrinsic uncoupling at constant ET capacity (LEAK respiration relative ET-capacity)
P/E OXPHOS control ratio	P/E OCR	succinate/FCCP	The limitation of OXPHOS capacity by the phosphorylating system
CI response ratio	CI-RR	ADP/DMP	ADP-stimulated response to malate and pyruvate, hypothesized to measure CI-linked phosphorylation
CI–CII response ratio	CI–CII-RR	(ADP-DMP)/(succinate-glutamate)	Hypothesized to measure alterations in either CI- or CII-linked phosphorylation relative to each other
N/NS pathway control ratio	N/NS PCR	glutamate/succinate	Relative N-pathway/CI-linked pathway function (in the coupled state)
S/NS pathway control ratio	S/NS PCR	rotenone/FCCP	Relative S-pathway/CII-linked pathway function (in the noncoupled state)

**Table 3:** The correlation between parameters of respiration in PBMCs, platelets and muscle fibers. CS = citrate synthase; CI = confidence interval; Other abbreviations, see Table 1. *PBMC and platelet respiration was normalized to cell count, muscle respiration was normalized to mass. ** Muscle fibers did not have digitonin added to them.

	PBMCs and platelets			PBMCs and muscle fibers			Platelets and muscle fibers		
	Pearson's r (95% CI)	p	n	Pearson's r (95% CI)	p	n	Pearson's r (95% CI)	p	n
Cell count-normalized*									
ROUTINE respiration	**0.14 (0.03–0.24)**	0.02	318	n/a	n/a	n/a	n/a	n/a	n/a
Background respiration**	0.07 (−0.04–0.18)	0.20	318	0.31 (−0.63–0.11)	0.15	24	0.28 (−0.62–0.14)	0.18	24
N-OXPHOS capacity(PM)	**0.13 (0.02–0.23)**	0.03	318	0.28 (−0.14–0.61)	0.19	24	0.04 (−0.37–0.44)	0.85	24
N-OXPHOS capacity	**0.13 (0.02–0.23)**	0.03	318	0.20 (−0.23–0.56)	0.36	24	0.02 (−0.39–0.42)	0.95	24
NS-OXPHOS capacity	0.06 (−0.05–0.17)	0.27	318	0.17 (−0.25–0.54)	0.43	24	0.17 (−0.26–0.53)	0.44	24
LEAK respiration	0.07 (−0.04–0.18)	0.21	318	**−0.44 (-0.72–-0.04)**	0.03	24	0.29 (−0.13–0.62)	0.18	24
NS-ET capacity	**0.15 (0.04–0.25)**	0.01	318	0.03 (−0.38–0.43)	0.88	24	−0.03 (−0.42–0.38)	0.91	24
S-ET capacity	0.09 (−0.02–0.20)	0.10	318	−0.16 (−0.53–0.26)	0.45	24	0.13 (−0.29–0.51)	0.55	24
CIV capacity	**0.20 (0.09–0.31)**	0.001	283	0.27 (−0.16–0.61)	0.21	23	−0.25 (−0.60–0.17)	0.23	24
**CS-normalized**									
ROUTINE respiration	−0.02 (−0.18–0.13)	0.75	162	n/a	n/a	n/a	n/a	n/a	n/a
Background respiration**	0.12 (−0.04–0.27)	0.13	162	−0.23 (−0.58–0.19)	0.28	24	**−0.45 (-0.72–-0.06)**	0.03	24
N-OXPHOS capacity(PM)	**0.19 (0.03–0.33)**	0.02	162	−0.34 (−0.08–0.65)	0.11	24	−0.08 (−0.47–0.34)	0.73	24
N-OXPHOS capacity	**0.20 (0.04–0.34)**	0.01	162	−0.21 (−0.56–0.21)	0.33	24	−0.40 (−0.69–0.00)	0.05	24
NS-OXPHOS capacity	**0.19 (0.04–0.34)**	0.01	162	−0.38 (−0.68–0.03)	0.07	24	**−0.46 (-0.73–-0.07)**	0.02	24
LEAK respiration	0.10 (−0.06–0.25)	0.23	162	−0.08 (−0.47–0.34)	0.72	24	0.04 (−0.37–0.43)	0.87	24
NS-ET capacity	**0.30 (0.15–0.43)**	<0.001	162	−0.04 (−0.44–0.37)	0.85	24	−0.39 (−0.69–0.02)	0.06	24
S-ET capacity	**0.22 (0.07–0.37)**	0.004	162	−0.18 (−0.55–0.24)	0.34	24	−0.31 (−0.63–0.11)	0.14	24
CIV capacity	**0.27 (0.11–0.41)**	0.001	141	0.40 (−0.02–0.70)	0.06	23	−0.14 (−0.52–0.28)	0.51	24
**Internal ratios**									
CI-RR	**0.14 (0.03–0.25)**	0.01	317	−0.40 (−0.69–0.01)	0.06	24	−0.01 (−0.41–0.39)	0.96	24
RCR	**0.16 (0.05–0.26)**	0.006	317	−0.38 (−0.68–0.03)	0.07	24	−0.02 (−0.42–0.39)	0.94	24
L/E CCR	**0.20 (0.09–0.30)**	<0.001	317	−0.05 (−0.44–0.36)	0.82	24	−0.01 (−0.41–0.39)	0.96	24
P/E OCR	**0.33 (0.23–0.42)**	<0.001	318	−0.19 (−0.55–0.23)	0.36	24	−0.37 (−0.67–0.04)	0.08	24
CI–CII-RR	**0.29 (0.18–0.38)**	<0.001	317	0.14 (−0.28–0.52)	0.51	24	0.14 (−0.28–0.51)	0.53	24
L/P CCR	**0.12 (0.01–0.23)**	0.03	317	−0.33 (−0.65–0.09)	0.12	24	0.23 (−0.19–0.58)	0.29	24
N/NS RCR	**0.42 (0.33–0.51)**	<0.001	318	0.31 (−0.11–0.64)	0.14	24	−0.03 (−0.43–0.38)	0.90	24
S/NS RCR	**0.33 (0.23–0.43)**	<0.001	318	0.16 (−0.26–0.53)	0.45	24	0.35 (−0.07–0.66)	0.10	24

**Fig. 1:**
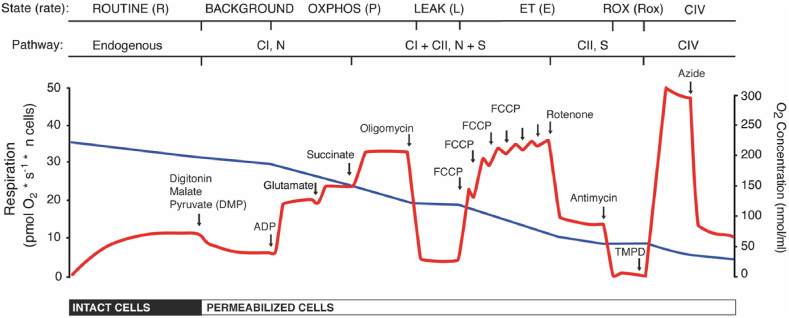
Study protocol. Illustration of the study protocol for permeabilized platelets and PBMCs. Additions of substrates, inhibitors and uncouplers to the chamber are indicated by the arrows. The first top bar illustrates respiratory states and corresponding rates (except “CIV”, which is not a respiratory state). The second top bar illustrates main substrate pathways and respiratory complex through which the electrons are donated. The gray line indicates the O_2_ concentration in the chamber, corresponding to the right y-axis. The black line indicates the O_2_ flux measured per n number of cells (n = 10^−8^ platelets, n = 10^−6^ PBMCs, respectively), corresponding to the left y-axis. Labels on the y-axes before the addition of ADP do not apply to the muscle fibre protocol. CI = complex I; CII = complex II; CIV = complex IV; N = NADH-linked substrates; S = succinate; OXPHOS = oxidative phosphorylation; ETS = electron transport system. ROX = residual oxygen consumption.

**Fig. 2:**
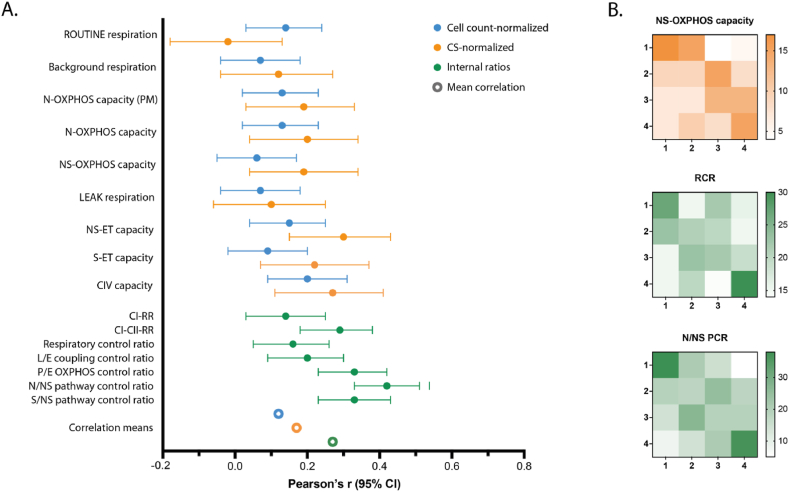
Platelet and PBMC correlations. **A** Comparison of different methods of normalization. The plot shows correlation between platelet and PBMC cell count normalized respiratory values (O_2_ * s^−1^ * 10^−8^ platelets and O_2_ * s^−1^ * 10^−6^ PBMCs; blue filled dots), the same values normalized to citrate synthase (CS) activity (orange filled dots) and a selection of internal ratios (green filled dots). Bars represent 95% confidence interval (CI). The hollow dots show mean correlation (without CI) for the respective groups corresponding in color. For n refer to Table 2. **B** Frequency distribution of respiratory parameters grouped as quartiles (1–4) to illustrate the association of values far from the median (quartiles 1 and 4, respectively) in comparison with values near the median (quartiles 2 and 3). The tables are crosstabulations of a CS normalized respiratory value (orange heat map) and a selection of ratios (green heat maps) for platelets (y-axes) and PBMCs (x-axes). The color of each square represents the overlap in absolute numbers for each quartile; darker color for higher overlap. The right-hand scale translates color intensity in each square into the absolute number of overlapping cases. X- and y-axes represent the quartiles of respiratory values for platelets and PBMCs. (For interpretation of the references to color in this figure legend, the reader is referred to the Web version of this article.)

In general, neither PBCM nor platelet respiration correlated with muscle fiber respiration and that pattern did not change by any method of normalization. Only three parameters out of 48 tested were found to correlate significantly and these correlations were negative (NS-LEAK-respiration correlated negatively between cell count-normalized PBMCs and mass-normalized muscle fibers, and CS-normalized background respiration and NS-OXPHOS capacity correlated negatively between platelets and muscle fibers). In accordance with the study plan, Spearman rank correlations were also tested for all parameters in Table 2, in general yielding slightly higher (but occasionally lower) correlation coefficients than the Pearson tests but no major difference in the general pattern (Supplementary Table 2).

Fig. 2B visualizes how the correlation between PBMCs and platelets is distributed over quartiles for three of the normalized measurements (the top one is CS-normalized and lower two are normalized using internal ratios). The overlap in the top and bottom quartiles (bottom right and top left squares, respectively) is bigger than the overlap in the second and third quartiles, indicating that when a value deviates further from the median it is more likely to do so across cell types.

### 2.3 Correlation between respiration and citrate synthase

**Fig. 3:**
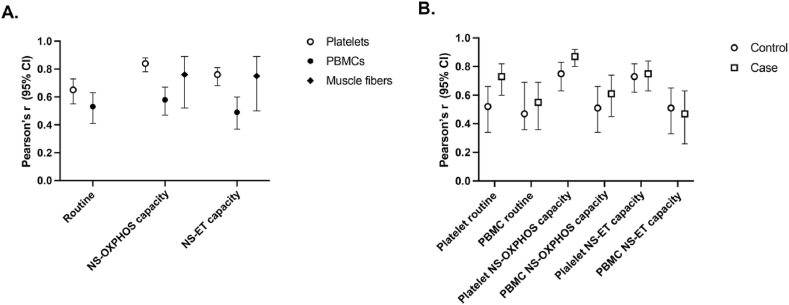
Correlations between respiration and citrate synthase activity. **A** Correlation between CS activity (μmol*min^−1^*ml^−1^), and basal, succinate and FCCP respiration (pmol O_2_*s^−1^*ml^−1^), for platelets (n = 163), PBMCs (n = 165) and between CS activity (μmol*min^−1^*mg^−1^) and the corresponding respiratory values (pmol O_2_*s^−1^*mg^−1^) in muscle fibers (n = 24). **B** The same PBMC and platelet correlations as in panel A, comparing patients (n = 84) and healthy controls (n = 73).

When examining the correlation between cell count-normalized respiratory values and CS activity in the same sample, there was generally a higher degree of correlation in platelet and muscle fiber samples compared to PBMCs (Fig. 3A). This finding was more prominent for NS-OXPHOS capacity and NS-ET capacity than for routine respiration and the former two were significantly higher in platelets as compared to PBMCs (Fishers r to z transformation, p < 0.001, Supplementary Table 5). The mean correlation for these two parameters were also higher in muscle, compared to PBMCs, but the differences were not significant. When comparing correlation between CS activity and respiration for healthy participants and patients there were no significant differences for PBMCs. Routine respiration and NS-OXPHOS capacity, but not NS-ET capacity, in platelets correlated slightly more with CS in patients (Fig. 3B–Supplementary Table 6).

### 2.4 Blood cell and muscle fiber alterations in athletes

**Fig. 4:**
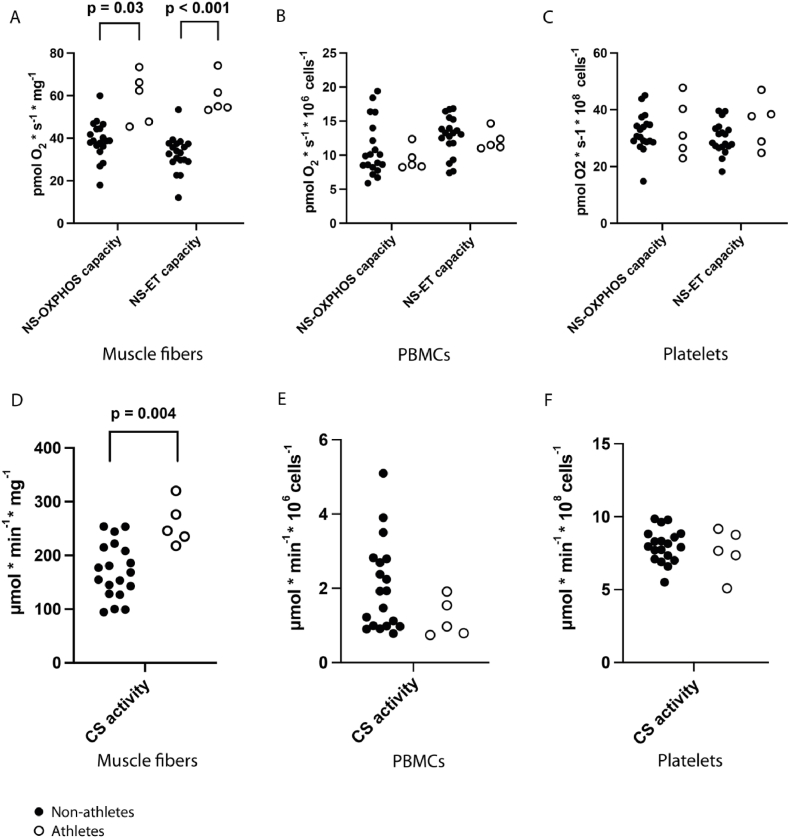
Respiration in a group of athletes compared to non-athletes. Significant group differences (P < 0.05 according to Mann-Whitney *U* test) are displayed. Black dots = non-athletes (n = 19). White dots = athletes (n = 5). Graphs A-C show mitochondrial respiration in **A** muscle fibers, **B** PBMCs and **C** platelets. Graphs D-F show citrate synthase (CS) activity in **A** muscle fibers, **B** PBMCs and **C** platelets.

Mass-specific mitochondrial respiration in muscle fibers in a group of five semi-professional athletes (n = 5, mean age: 25 years, 2 females/3 males) compared to a control group of non-athletes (n = 19, mean age: 26 years, 8 females/11 males) was significantly higher (Fig. 4A) but such differences were seen neither in PBMCs nor in platelets (Fig. 4B–C). The muscle fibers from the athletes had significantly higher CS activity (Fig. 4D) as opposed to CS activity in blood cells, which was not affected in the athletes (Fig. 4E–F). When normalizing respiration to CS, the differences in respiration were eliminated in athletes and remained absent in the blood cells (Supplementary Table 7).

### 2.5 Blood cell alterations in patients with mitochondrial disease

**Fig. 5:**
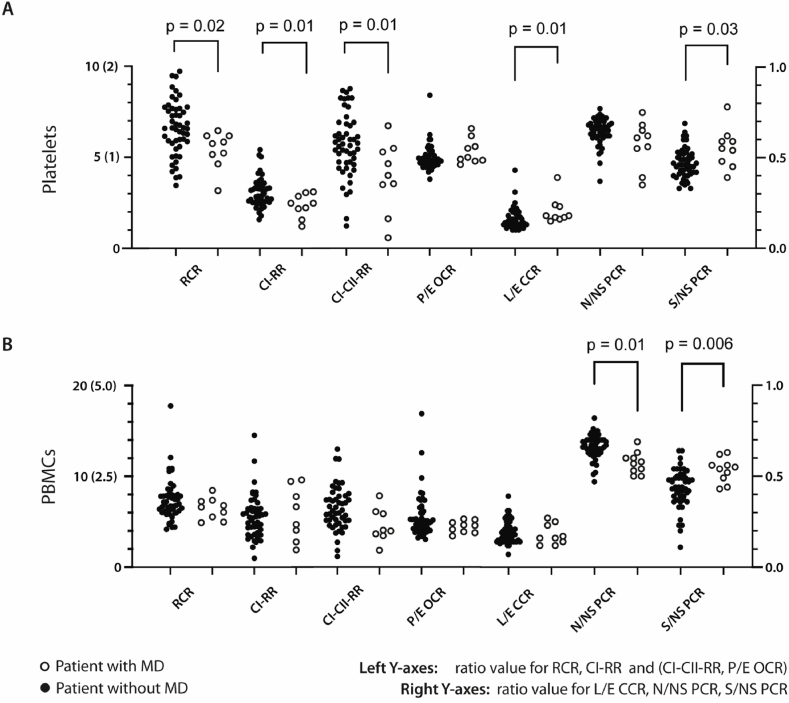
Respiration in in a group of pediatric patients with mitochondrial disease compared to other pediatric patients. Significant group differences (P < 0.05 according to Mann-Whitney *U* test) are displayed. For abbreviations, see Table 1. **A** Comparison of internal ratios in platelet mitochondria from pediatric patients without mitochondrial disease (left side dots, n = 50) and patients with confirmed mitochondrial disease (right side dots, n = 9). Left y-axis shows the scale of RCR and CI-RR (0–10) and in parenthesis for CI–CII-RR and P/E OCR (0–2). Right y-axis shows the scale for the remaining ratios (0.0–1.0). **B** Corresponding analysis in PBMCS from patients without mitochondrial disease (n = 50, except CI–CII-RR n = 49) and with mitochondrial disease (n = 9, except CI-RR n = 8). Left y-axis shows the scale of RCR and CI-RR (0–20) and in parentheses for CI–CII-RR and P/E OCR (0–5.0). Right y-axis shows the scale for the remaining ratios (0.0–1.0).

Platelets and PBMCs from nine pediatric patients with mitochondrial disease were compared to samples from another group of pediatric patients where mitochondrial disease was initially included in the differential diagnosis. (The patients in this analysis are a subset of the patients presented in a previous publication [13]. The subset comprises all cases where both platelet and PBMC data were available. For more information on the individual mitochondrial diagnoses we refer to Table 3 of that publication; the patients included in this study are the cases indexed as 2, 3, 5, 6, 10, 11, 13, 14 and 15 [13].) Several of the respiratory ratios were significantly altered in the group with mitochondrial disease, mainly in platelets (Fig. 5A, 5/7 ratios) but also in PBMCs (Fig. 5B and 2/7 ratios) (see also Supplementary Table 8).

## 3 Discussion

In this study, within and across tissue correlations of mitochondrial respiration were examined, to further the understanding of blood cell respiration as a biomarker for less accessible tissues or systemic metabolic alterations. The main findings were that correlation within blood tissue was generally statistically significant, but weak, while neither platelets nor PBMCs correlated with muscle fibers. However, additional analyses provided nuances to these largely negative findings.

While the majority of the correlations in our main analysis between PBMCs and platelets were weak or very weak, they were nonetheless significant and, as expected, normalizing to CS or normalizing by the use of internal ratios increased both the correlation coefficients and the significance level (in several cases to the <0.001 level). One potential source of error for the correlations is the impurity of the blood samples inherent to the isolation method. The potential effect is difficult to estimate but we did measure mean contamination levels, which in the PBMC samples was 4 platelets (SD ± 3, n = 308) per PBMC and in the platelet samples 37 PBMCs (SD ± 42, n = 305) per 10 000 platelets, numbers which are common according to recent methodological literature [1,22]. Contaminations in PBMCs and platelets likely make the correlations inaccurate, but without additional experiments it is difficult to calculate their exact contributions to respiration.

Our main results do not support the hypothesis that both blood cell types correlate strongly with muscle as they correlate weakly with each other. To our knowledge there is no previous study on PBMCs and platelets of this size but the conclusion is compatible with previous work comparing blood cell respiration with muscle. Two studies comparing PBMCs with muscle did not find any correlation [10,11] and the correlations that have been found were found in platelets or monocytes [8,9,11].

In this study neither PBMCs nor platelets correlated with muscle except for very few negative correlations, to which no other plausible explanation outside of statistical noise have been found. The general lack of correlation is compatible with our hypothesis that correlations across tissues would be smaller than within the same tissue but should still be interpreted very cautiously since the sample size of the subgroup with muscle biopsies (n = 24) was much smaller than the main study population (n = 318). To conclude a real lack of correlation could be to inflict a type II error. The negative results may not be able to settle the question of whether blood cells can be used as biomarkers for the bioenergetic profile of muscle fibers, but they add disfavor to the conflicting results of previous publications [[8], [9], [10], [11]]. It should be noted that the conflicting results were produced by study populations differing substantially in their characteristics and that the population most similar to the one in this study, the young healthy males examined by Hedges et al., had the most negative results [10]. Muscle samples from a more widely representative study population or a separate one with children would have expanded the applicability of the results but the latter was not feasible for this study for logistical and ethical reasons.

Furthermore, when Hedges et al. failed to reproduce the significant monocyte-muscle correlations of Tyrell et al. using PBMCs they noted that their samples contained only a small fraction of monocytes and that these cells may be better suited as biomarkers than other mononuclear cells [9,10]. Differential count on a subset of PBMC samples (n = 251) in our study revealed that monocytes in average comprises at most 7.4% of the analyzed cells, leading us to the same assessment. Mean lymphocyte count was 85.6% (SD ± 9.2). Metabolic changes in mitochondria are highly involved in hematopoietic stem cell differentiation and previous research has indicated that different peripheral blood cells and platelets have distinctly different bioenergetic profiles [23,24].

The importance of differing bioenergetic profiles was further explored in Fig. 3. The correlation between cell count-normalized – or in the case of muscle fibers, mass-normalized – respiration and CS activity was examined. CS activity is an often used and reliable marker of mitochondrial content [25]. We assumed that stronger correlation between CS and respiration values would indicate a more reliable assessment of mitochondrial function. Platelets and muscle fibers had higher correlation with CS activity than did PBMCs. The differences in degree of correlation were only significant for platelets but the lack of significance for muscle fibers could be due to the small sample size (Fig. 3A–Supplementary Table 5). The NS-OXPHOS and NS-ET capacities correlated stronger than routine respiration, which was expected, as the maximum coupled and uncoupled capacities are limited by mitochondrial content whereas routine respiration is not (it is limited by, among other things, the amount of endogenous substrates). The correlations with CS activity were moderate to strong both in healthy participants and patients (Fig. 3B).

In a subgroup of the participants, we compared semi-professional athletes to non-athletes on a group level. NS-OXPHOS and NS-ET capacities per mass unit of muscle fiber were significantly higher for the athletes (Fig. 4A). CS activity was also significantly higher (Fig. 4D) in athletes compared to non-athletes. Since normalizing the respiratory values to CS eliminated the significant differences in respiration (Supplementary Table 7), it is likely that the increased respiration in the muscles of the athletes was related to increased mitochondrial content (measured as CS activity). No differences between athletes and non-athletes in respiration or mitochondrial content were detected in either blood cell type (Fig. 4B–C, E-F, Supplementary Table 7). The results in muscle agree with previous research [26,27]. Much less is known about exercise-induced changes in blood cell respiration. Recent studies have detected increased fatty-acid dependent respiration in PBMCs and increased LEAK state respiration in platelets as acute post-exercise effects [28,29]. We are not aware of any previous study on permanent exercise-induced changes in the mitochondrial content of blood cells.

In another subgroup analysis, blood cells in a group of pediatric patients with confirmed mitochondrial disease were compared to patients that were initially suspected to have mitochondrial disease but without a final diagnosis. As is shown in Fig. 2B, there is higher across-sample overlap for values deviating further from the average and this supports the theory that conspicuous respiratory dysfunction – such as mitochondrial disease – is more likely to manifest itself across tissues than do slight alterations. Since our analysis showed that internal ratios increased the correlation as well as, or better than CS normalization, we only examined the ratios. (This method of normalization was also used in our previous related work on patients with mitochondrial disease [13].) On a group level, the comparisons showed significant differences in several ratios. These findings are in line with previous research, as group level differences in mitochondrial respiration in blood cells or platelets for patients with MD has previously been shown in small samples [[30], [31], [32]]. Most differences were seen in platelets, but the N/NS PCR was significant only in PBMCs and not platelets.

Strengths of this study include a large study population used in the main analysis, which together with the precision of high-resolution respirometry enabled the detection of weak correlations. Another strength of the study is that citrate synthase normalization was performed for a large subset of the main population.

A major limitation of the study is that it is mainly a retrospective, single-center study. Power calculations were not possible. Though we believe the study population as a whole is well-balanced and diverse enough for the purposes of the analyses, it is inevitable that the retrospective nature of the study and non-randomized selection of participants, including selection for several sub-analyses that were made on the basis of available data (CS-normalization, muscle correlation, assessment of CIV-activity), introduce possible bias.

Another major limitation of this study is the lack of adjustment for important contributors to the correlation, such as age, sex, and metabolic disease. It is possible that several such factors contributed to the correlation found, but adjusting for them would more likely lower than raise the already weak correlation. Such factors are sometimes adjusted for in a multivariate regression model, but regression was not performed here since the causality condition was not believed to be satisfied for these data (i.e. that one variable is clearly dependent on the other). While it might have been possible to use partial correlation to single out some individual factors contributing to the correlation found, we opted instead to present the results of several relevant subgroups, among which some differences can be noted, in Supplementary Tables 3 and 4

Data on BMI, smoking status, medications and psychological comorbidities, which would also have been interesting denominators, were lacking for most subjects. For the comparison of athletes versus non-athletes, it would have been desirable to be able to present more detailed data, such as exercise regimens. On the other hand, the highly significant differences seen in the muscle comparisons indirectly confirm a successful selection of participants into the two groups. The fact that intact study protocols were not included may be a limitation of the study but the permeabilized protocol was preferred as it was assumed that the maximal capacities (NS-OXPHOS and NS-ET) would correlate best. Digitonin and saponin, which are used to permeabilize cell membranes, introduce a possible risk of mitochondrial toxicity that is not present in the intact protocols but this risk can be kept low by using validated concentrations. Another limitation was that the results could not be adjusted for differential count, this might have strengthened correlations.

In conclusion, the weak within tissue correlation coupled with the lack of across-tissue correlation found in this study argues against the notion that blood cell respiration can be used as accurate markers for muscle respiration in a general population. However, additional results support the hypothesis that blood cells, especially platelets, may reflect certain, pronounced metabolic alterations in less-accessible tissues or systemically in the body.

## 4 Methods

### 4.1 Study population

Blood samples were collected from patients and healthy volunteers including both sexes at a wide age span. Most samples were collected as part of other studies and platelet and PBMC data from the participants have been published in other contexts [[13], [14], [15], [16], [17], [18], [19], [20]]. Some samples, including all muscle biopsies, were collected principally for this study. There were 318 participants in total, of which 226 were patients, with diseases such as neurodegenerative diseases, pediatric neurological conditions and sepsis, and 92 were healthy volunteers. Out of the 318 participants, 82 were children. PBMCs and platelets were analyzed in all 318 participants. In 24 of the adult participants, muscle biopsies were obtained in addition to the blood samples.

### 4.2 Sample preparations

Venous blood was collected in K2 ethylene diamine tetraacetic acid (EDTA) tubes. Blood cells were separated through consecutive centrifugations and further prepared as described in previous publications and analyzed within three to 5 h [16,19]. The EDTA tubes were centrifuged 15 min at 300 g to yield a platelet rich plasma (PRP), which was pipetted and re-centrifuged for 5 min at 4600 g. This yielded a layer of close to cell free plasma and a platelet pellet. The pellet was dissolved in 1–3 ml of the study participant's own plasma by gentle pipetting to obtain a highly concentrated PRP [16]. The PBMC preparations were made from the remaining blood, after the first PRP had been pipetted off following the initial centrifugation (see above). This blood was diluted in saline (NaCl 9 mg/ml) up to 6 ml and carefully added to tubes containing 3 ml Lymphoptep™, which were centrifuged for 25–30 min at 800 g. The mononuclear cell layer and lymphoprep fraction were collected and washed in saline (∼10 x dilution), then recentrifuged for 10 min at 250 g. The supernatant layer was aspirated and resuspended in 0.5–1 ml the participant's own plasma. All centrifugations described above were made at room temperature [19].

Muscle biopsies were obtained under sterile conditions after a subcutaneous injection of 5 ml mepivacaine/norepinephrine (10 mg/ml + 5 μg/ml) through a 14 gauge needle in m. vastus lateralis. The local anesthetic was applied carefully not to expand down to the muscle, as local anesthetics are known to effect mitochondrial function [28]. A sample of approximately 20 mg tissue was taken directly under the muscle fascia and was immediately transferred to an ice-cold biopsy preservation solution (BIOPS; 10 mM Ca-EGTA buffer, 0.1 μM free calcium, 20 mM imidazole, 20 mM taurine, 50 mM K-MES, 0.5 mM DTT, 6.56 mM MgCl_2_, 5.77 mM ATP, 15 mM phosphocreatine, pH 7.1). The sample was then dissected under a microscope using forceps to mechanically separate fibers and to remove fat and connective tissue. The fiber bundles were permeabilized for 30 min in 2 ml BIOPS + Saponin (20 μl of 5 mg/ml) and afterwards washed in MiR05 (0.5 mM EGTA, 3 mM MgCl_2_, 60 mM k-lactobionate, 20 mM taurine, 10 mM KH_2_PO_4_, 20 mM HEPES, 110 mM sucrose, 1 g/l BSA, pH 7.1) [33] for another 10 min. Before respiratory analyses, the biopsy wet weight was measured with a digital precision scale (Precisa 40SM-200A).

The contents of the chamber (cells/fibers in MiR05; 2 ml) were frozen and stored at −80 °C after each experiment and later thawed on ice for citrate synthase (CS) measurements. CS activity was measured, as previously described, using a commercially available kit in accordance with the manufacturer's instructions (Citrate Synthase Assay Kit, CS0720, Sigma–Aldrich, St Louis, MO, USA) [14]. Samples were sonicated (PBMCs 30 s, platelets and muscle fibres, 2 × 60 s) and transferred onto a 96 well plate mixed with assay buffer, acetyl-CoA and 5,5′-Dithiobis-(2-nitrobenzoic acid) (DTNB). The optimal sonication times were determined in a different set of experiments (data not shown). A spectrophotometer set to 412 nm recorded the absorbance before and after the addition of oxaloacetic acid, as the formation of 5-thio-2-nitrobenzoic acid (TNB) can be used to estimate the activity of CS. The absorbance was followed for 2 min and the activity of the sample was calculated using the extinction coefficient of TNB, which is 13.6 mM^−1^ ⋅ cm^−1^ at 412 nm.

### 4.3 High-resolution respirometry and respiratory ratios

Mitochondrial respiration was measured at 37 °C in high-resolution oxygraphs (Oxygraph-2k Oroboros Instruments, Innsbruck, Austria). The experiment design for blood components has previously been published in detail and was identical for platelets and PBMCs, except that the software (DatLab Software 4.3, Oroboros Instruments, Innsbruck, Austria) was set to record respiration per 10^8^ platelets and 10^6^ PBMCs respectively to make graphic comparisons more convenient [16].

In summary, the cells were suspended in an airtight chamber, in MiR05 [33], and changes in the rate of oxygen consumption (O_2_ flux) were measured with a high precision sensor following subsequent additions of substrates, uncouplers and inhibitors to assess different pathways and other aspects of mitochondrial respiration. This type of protocol is called substrate-inhibitor-uncoupler-titration (SUIT) [34].

The details of the study protocol are outlined in Fig. 1. The graph illustrates a typical experiment; all measurements were made consecutively in the same sample. Mitochondrial respiration was categorized by the respiratory state of the mitochondria (first top bar) and by substrate pathways (second top bar).

Initially, routine respiration was measured in the intact cells after reaching a stable plateau. Digitonin (6 μg per 10^6^ PBMCs, 1 μg per 10^6^ platelets) was added to permeabilize the cell membrane, after which malate (5 mM) and pyruvate (5 mM) were added followed by ADP (1 mM) to induce oxidative phosphorylation (OXPHOS). At this point electrons are provided to complex I of the electron transfer system (ETS) by the electron carrier NADH, which is why the measured respiration can be said to represent the N-linked pathway. The addition of glutamate (5 mM) provides further electrons via NADH. The addition of succinate (10 mM) provides additional electrons via complex II, also known as the S-linked pathway.

The combined pathways (N + S) were measured and represent the maximal coupled respiration, known as the NS-OXPHOS capacity (or alternatively just ‘OXPHOS capacity’, without the prefix). Next, the ATP synthase inhibitor oligomycin (1 μg/ml) was added, inhibiting OXPHOS and allowing the measurement of LEAK respiration (LEAK) over the inner mitochondrial membrane.

This was followed by titration of the uncoupler protonophore carbonyl cyanide 4-(trifluoromethoxy) phenylhydrazone (FCCP), to uncouple the ETS from ATP synthesis and the measurement of respiration at the titration plateau represents the maximal uncoupled respiration, known as the NS-ET capacity (or alternatively just ‘ET capacity’).

Subsequently, the complex I inhibitor rotenone (2 μM) was added to measure the S-linked pathway separately from the combined pathway. The complex III inhibitor antimycin A (1 μg/ml) was added after this to adjust for residual oxygen consumption in the cells. This O_2_ flux was subtracted from all previous measurements before further calculations were made.

In select cases, tetramethylphenylenediamine (TMPD, 0.5 mM) followed by sodium azide (10 mM) were added at the end of the experiment to assess the activity of complex IV. The addition of ascorbate was tested (data not show) in a prototype version of the protocol but caused too much auto-oxidation and was omitted from the protocol. (This was an unexpected finding, as the purpose of ascorbate is to keep TMPD in a reduced state. The auto-oxidation was likely catalyzed by different metal-containing proteins in the platelet samples. TMPD without ascorbate in platelets was shown to give reproducible results [16].) Ascorbate was likewise omitted from the PBMC and muscle preparations to harmonize the protocols.

The muscle fiber protocol was similar to the protocol for platelets and PBMCs, except for a few details: Respiration was recorded per mg of muscle tissue. Digitonin was not added, as the fibers were saponin-permeabilized during preparation. ADP was given at a dose of 2 mM instead of 1 mM and oligomycin in the dose of 2 μg/ml instead of 1 μg/ml. The muscle respirometry was performed in two chambers for each individual and mean values were calculated. Respiratory values were corrected not for antimycin but for respiration before adding substrates (malate, pyruvate) and ADP, since this was assumed to represent residual oxygen consumption (ROX) more reliably than the levels after the addition of antimycin A.

Table 2 explains the terminology used in text, tables and figures, and its relation to the additions and measurements made during the experiment. For a more comprehensive description of the states, rates and pathways we refer to other literature [34]. In addition to normalizing for cell count and CS, we have normalized values by calculating a number of internal ratios, which are further explained in Table 2. Two of the ratios (CI response ratio, CI–CII response ratio) are not in common use but are based on our previous publication where they displayed a promising diagnostic accuracy for primary mitochondrial disease [13].

### 4.4 Statistics

Correlation between platelets, PBMCs and muscle fibers was examined for cell count-normalized values (or, in the case of muscle fibers, mass-normalized values), CS-normalized values and internal ratios using Pearson correlation. As a sensitivity measure, we also analyzed Spearman rank correlation to exclude any large discrepancies. Mean correlations for cell count-normalized and CS-normalized values, and internal ratios respectively were calculated to ease visual comparison in the diagram. To analyze whether extreme respiratory values correlated better than average values we compared quartile overlap for selected parameters and ratios.

As a quality control of the measurements of mitochondrial respiration we analyzed the correlation between CS values (as a measure of mitochondrial content) and respiration in each sample, comparing the sample types (PBMCs, platelets, muscle fibers) to one another, and, for platelets and PBMCs, comparing healthy participants with patients. For assessing the significance of the difference between two correlation coefficients the Fisher r-to-z transformation was applied.

In a subgroup of the healthy participants we made groupwise comparison of athletes and non-athletes, examining respiration and citrate synthase activity in muscle, platelets and PBMCs.

Finally, we compared platelet and PBMC respiration on a group level between pediatric patients with confirmed mitochondrial disease to pediatric patients without mitochondrial disease, using internal respiratory ratios.

To test for statistical significance in the analyses concerning athletes and mitochondrial disease patients respectively, the Mann-Whitney *U* test, was selected due to small and unequally distributed sample sizes in the groups compared.

In addition to the analyses mentioned above, descriptive blood cell and muscle fiber respirometry values (mean values for cell-count or mass normalized respiration, and CS-normalized respiration) for the adult participants, both for the whole group and for the healthy adults separately, were calculated.

Statistical significance was defined as p < 0.05 for all analyses. For the correlation coefficiants (Pearson's r or Spearman's rho), we defined 0.00–0.19 as very weak, 0.20–0.39 as weak, 0.40–059 as moderate, and 0.60–0.79 as strong and 0.80–1.00 as very strong correlation.

### 4.5 Study approval

This study has been approved by the regional ethical review board of Lund, Sweden (113/2008, 59/2009, 97/2009, 89/2011, 320/2011) and the scientific ethical committee of Copenhagen County, Denmark (H–C-2008-023). Written informed consent was obtained from each participant or from the participant's parent or guardian or, in applicable cases, from other relative.

## CRediT authorship contribution statement

**Emil Westerlund:** Writing – review & editing, Writing – original draft, Visualization, Validation, Software, Project administration, Methodology, Investigation, Formal analysis, Data curation, Conceptualization. **Sigurður E. Marelsson:** Writing – review & editing, Investigation, Formal analysis. **Michael Karlsson:** Writing – review & editing, Methodology, Formal analysis, Data curation, Conceptualization. **Fredrik Sjövall:** Writing – review & editing, Methodology, Formal analysis, Data curation. **Imen Chamkha:** Writing – review & editing, Validation, Methodology, Formal analysis, Data curation. **Eleonor Åsander Frostner:** Writing – review & editing, Validation, Project administration, Methodology, Investigation, Formal analysis, Data curation. **Johan Lundgren:** Writing – review & editing, Formal analysis. **Vineta Fellman:** Writing – review & editing, Formal analysis. **Erik A. Eklund:** Writing – review & editing, Formal analysis. **Katarina Steding-Ehrenborg:** Writing – review & editing, Formal analysis, Data curation. **Niklas Darin:** Writing – review & editing, Formal analysis. **Gesine Paul:** Writing – review & editing, Formal analysis. **Magnus J. Hansson:** Writing – review & editing, Methodology, Investigation, Formal analysis, Data curation, Conceptualization. **Johannes K. Ehinger:** Writing – review & editing, Writing – original draft, Supervision, Software, Resources, Methodology, Investigation, Formal analysis, Data curation, Conceptualization. **Eskil Elmér:** Writing – review & editing, Writing – original draft, Supervision, Resources, Project administration, Methodology, Investigation, Funding acquisition, Formal analysis, Data curation, Conceptualization.

## Declaration of competing interest

Imen Chamkha, Johannes K. Ehinger, Eskil Elmér, Magnus J. Hansson, Michael Karlsson, and Eleonor Åsander Frostner have equity interests in, and/or have received salary support and/or travel reimbursements and/or grants from Abliva AB (formerly NeuroVive Pharmaceutical AB), a public company developing pharmaceuticals in the field of mitochondrial medicine. The other authors declare no financial or commercial conflict of interest.
